# Laparotomies Performed during the past Year, (Opie.)

**Published:** 1892-03

**Authors:** W. E. B. Davis

**Affiliations:** Formerly Surgeon to the Birmingham Hospital of United Charities; President of the Tri-State Medical Society of Alabama, Georgia and Tennessee; Secretary of the Southern Surgical and Gynecological Association, etc., etc. Birmingham, Ala.


### "LAPAROTOMIES PERFORMED DURING THE PAST YEAR. (OPIE.)"*

### A DISCUSSION ON DR. THOMAS OPIE'S PAPER AT THE FOURTH ANNUAL MEETING OF THE S. S. AND G. ASSOCIATION.

BY W. E. B. DAVIS, M. D., †

Formerly Surgeon to the Birmingham Hospital of United Charities; President of the Tri-State Medical Society of Alabama, Georgia and Tennessee; Secretary of the Southern Surgical and Gynecological Association, etc., etc.

I enjoyed listening to the paper by Dr. Opie, from the fact that he laid stress on his fatal cases. I think we have all read enough about ten or twenty successful sections, etc., in which the title of the paper is more of an advertisement for the surgeon than for the benefit of those who read the paper. I do not mean anything personal; but I do really believe, and I have expressed it recently, that the habit of reporting a series of cases has gotten to be a great evil. There is a tendency in reporting these cases to make it appear that they all get well without complications ; in other words, there is a tendency in the profes-

*See p. 577, December Issue.

†These notes were too late for earlier insertion.—ED.

sion, especially among those who have hospitals, and I do not refer to any particular man in this association or outside of it, to report successful cases, which are a good advertisement for the surgeon. There is often a motive in reporting a series of cases, and we can see how it might do a man's practice good when it would not materially benefit medical literature. But Dr. Opie has emphasized his fatal cases, and I congratulate him upon the work he has done. We learn a great deal from the publication of such cases.

There were one or two points referred to that I desire to speak of, and the first is with reference to drainage. When we see and read of the successful results of men who seldom use drainage, we feel that we ought not to use it. We know that Martin and others rarely use drainage and that their patients do well, yet I do not believe we can draw any lesson from that. The surgeon who hardly ever uses a drainage tube, if he should use it, does not take care of the tube like the man who resorts to it in the majority of cases.

I think another mistake is made in the size of the tube. To put a large tube in the abdomen is a thing we ought not to do. The small tubes, such as are used, will give us sufficient drainage; usually they can be removed in from twelve to twenty-four hours. The cases in which drainage is used get well with fewer complications. About germs in the abdomen, I believe with other speakers that the peritoneum can take up a number of pathogenic germs, if it is kept in good condition. If properly drained, the peritoneum will be better capable of digesting or taking up and destroying germs. A peritoneum in which there is no drainage would succumb to very few microbes.

In reference to Dr. Johnson's case of general suppurative peritonitis, I want to say that where we have a quart or gallon of pus in the abdomen and recovery follows, it is not due to general suppurative peritonitis, but it is due to a localized peritonitis or abscess. If we get general suppurative peritonitis, where the pus is due to peritonitis proper, I do not think there is any hope for a patient to recover. This has been demonstrated by gunshot wounds of the abdomen, and I have seen quite a number of cases. I agree with Gravitz and others, that by the time a general peritonitis becomes purulent you have lost all opportunity of saving your patient. For instance, if a man is shot in the abdomen, and twenty-four hours elapse before you see him, it would be better surgery to refuse to operate. It is better to teach practitioners that if we operate on these cases we must do so early, and if we are not allowed to do so, it is bad surgery to operate late. We bring surgery into disrepute and thus fail to operate on many patients whose lives we might save by early operations.

In reference to getting rid of pus, I am inclined to the idea of flushing the cavity, notwithstanding the fact that I know pus can be sponged out without the flushing of the cavity. The general surgeons, who are doing operative work for disease of the appendix and abscesses around its base, as McBurney, Stimson and others, sponge pus out of the cavity, then pack with gauze, and the patients get well. However, there is not the danger from general infection, that we have been led to believe, in flushing the cavity. We do not have as high a temperature ; we do not have as much traumatism, and we relieve shock ; and then by putting in a small drainage tube our patients get along all right.

